# Specialization and Injury Risk in Different Youth Sports: A Bio-Emotional Social Approach

**DOI:** 10.3389/fpsyg.2022.818739

**Published:** 2022-03-16

**Authors:** Teresa Iona, Simona Raimo, Daniele Coco, Patrizia Tortella, Daniele Masala, Antonio Ammendolia, Alice Mannocci, Giuseppe La Torre

**Affiliations:** ^1^Department of Surgical and Medicine Science, University of Magna Graecia, Catanzaro, Italy; ^2^Department of Education, Roma Tre University, Rome, Italy; ^3^Department of Education, Free University of Bolzano, Bolzano, Italy; ^4^Faculty of Economics, Universitas Mercatorum, Rome, Italy; ^5^Department of Public Health and Infectious Diseases, University of Sapienza, Rome, Italy

**Keywords:** injury risk, sport specialization, youth sports, adolescent, educational approach

## Abstract

**Aims:**

Sport specialization is an actual trend in youth athletes, but it can increase injury risk. The aim was to determine the eventual correlation between sports specialization and injury risk in various sports, using a biopsychosocial approach.

**Methods:**

169 sport-specialized athletes completed [(38 female, 131 male); overall (11.2 ± 2.7 years), (56.28 ± 15.72 kg), (161.3 ± 15.52 cm)] a self-reported questionnaire regarding sociodemographic (age, gender, educational level), physical-attitudinal, injuries and psychological-attitudinal To analyze data univariate and correlate analyses were used.

**Results:**

Of 169 athletes enrolled, 53% were single-sport specialized (reported participation in one sport and trained > 8 months/year). In team sports (100%, OR = 0.75; *p* = 0.022) a high risk of having to remain at rest for up to 1 month because of overuse was observed. Males who suffered direct trauma (70%; OR = 1.03; *p* = 0.006) in the team group (95%, OR = 0.09; *p* = 0.008) were more exposed to that type of injury, and also the type of specialization figured significantly (*p* = 0.047). In addition, interoceptive awareness correlates with injury (95%, 1.04, *p* = 0.01). This study shows that, even though young athletes seem to be at a high risk of becoming injured, early team sport specialization and a high performance level cannot be considered to be the only risk factors.

## Introduction

Sport specialization is an actual trend among young athletes, but it can increase the risk of injury (Pasulka et al., 2017; Post et al., 2017). This sport specialization refers to a year- or nearly year-round commitment to one sport to the exclusion of others (Jayanthi et al., 2013), and is becoming increasingly prevalent among pre-adolescent and adolescent athletes (McGuine et al., 2017).

A consensus-based conceptual definition for sport specialization has been developed using a Delphi method in which a group of multidisciplinary experts participated. The final consensus definition is as follows: *Sport specialization is intentional and focused participation in a single sport for a majority of the year that restricts opportunities for engagement in other sports and activities* (Bell et al., 2021). Social data indicated that approximately half of adolescent athletes participated on club teams in addition to their school-based teams year-round in a chosen sport. Recent author have reported prevalence rates of sport specialization among youth athletes of 17 to 41% (Bell et al., 2018; Post et al., 2018). This trend could be driven by a large number of factors such as an overall decrease in unstructured physical activity (i.e., “free play”), an increase in structured activity among youth (Somerset and Hoare, 2018), increased pressure on young athletes to excel in sport (Feeley et al., 2016) but also the potential economic benefit of success in sports (e.g., college scholarships, elite achievement, or high professional sports salaries) (Mosher et al., 2022).

As a result, there is concern that there will soon be more young athletes becoming specialized at an earlier age (Emery and Pasanen, 2019), and this could lead to negative effects in these athletes, including psychological burnout and an increased risk of musculoskeletal injuries (Popkin et al., 2019). Specialized athletes typically engage in a large amount of sport-specific training throughout the year, which is intensive, often technical or otherwise specific (Myer et al., 2015; LaPrade et al., 2016). The notion that earlier specialization increases the likelihood of eventually achieving elite sport performance mainly comes from research using the deliberate practice framework supported by the amount of practice was the most critical factor to becoming a master or an expert. The idea of deliberate practice and early specialization is based on the assumption that this is superior to deliberate play and involvement in (multiple sport during childhood for elite performance (Ericsson et al., 1993; Cote et al., 2011). As a result, sport specialization has been associated with an increased risk for injuries of overuse. Training volume is thought to be important, because intense, year-round participation in the sport of choice is a particularly concerning factor of specialization (Arnold et al., 2017). Training volume recommendations commonly cited in literature relate to months of the year and hours of organized sports participation per week. It is recommended that children and youth who participate in organized sports should not practice the same sport for more than 8 months a year, no more hours per week than their age (i.e., an athlete of 13 years of age should not participate in organized sports for more than 13 h per week) and no more than 16 h per week in total (Brenner and Council on Sports Medicine and Fitness, 2016). Very little research is concerned with the influence of the emotions on injuries in young and specialized athletes. There are at least a dozen models that try to establish a connection between psychological antecedents and the occurrence of sports injuries. In fact, personality, anxiety, hardiness, life event stress, achievement motivation and also interoceptive awareness (IAw) all seem to be particularly related to injury outcome (Kalkhoven et al., 2020). Interoception has been classically conceived to be the maintenance of an optimal physiological balance in the body—the homeostasis of the physiological system—through autonomic, neuroendocrine, and behavioral responses. Interoception also means one’s sense of the physiological condition of the body, and provides a basis for subjective feelings and emotions (Craig, 2003). Many studies have suggested that individuals having a stronger interoceptive ability report more intense emotions (Murphy et al., 2019), place more emphasis on the dimension of arousal in reporting their emotional experience (Barrett and Simmons, 2015), and manifest a stronger link between their bodily reactions to emotional stimuli and their subjective arousal ratings (Raimo et al., 2021).

The aim of the present study was to determine whether there is an association between the risk of injury in young athletes (7–18 years of age) and factors related to specialization, IAw and the physical well-being of young athletes.

## Materials and Methods

### Participants

A cross-sectional study was conducted. The sample was composed of young athletes who practiced various individual and team sports including basketball, volleyball, soccer, taekwondo and judo. These volunteer athletes were from Southern Italy and were recruited during tournaments, competitions and summer training sessions using the university stakeholders’ mailing list, numerous coaches, health professionals, sports medicine doctors and physiotherapists. All subjects participated on a voluntary basis and signed an informed consent before being accepted for the study. Eligible participants were between the ages of 7 and 18 and were those who had practiced organized sports during the previous 12 months. Parental consent was obtained for each participant under the age of 18. The study was approved by the Local Ethics Committee and was performed in accordance with the Declaration of Helsinki.

### Measurements

A questionnaire filled out by each athlete was used for data collection. This tool had been composed with the participation of an interdisciplinary team, including orthopedic surgeons, physical therapists, athletic trainers, training academy instructors and epidemiologists.

#### Specialization and Risks of Injuries Testing

Participants completed a sport specialization survey and a lower extremity injury history survey. The questionnaire consists of following sections: sociodemographic (age, gender, educational level), injuries (recall and description) and training specialized information (months a year of participation in the primary sport and in organized sports in total; average weekly hours of participation in the primary sport during the first sporting season; total average weekly hours in organized sport, average weekly hours in unorganized sport [gymnastics lessons, playing with friends, etc.) and competitions (national, international)]. Based on their responses, the athletes were classified according to their self-described level of specialization (high, medium, low) (Pasulka et al., 2017), and type of sport practiced (individual and/or team) (Stracciolini et al., 2014). The Classificazione degli Infortuni per Giovani Atleti Specializzati—CIGAS questionnaire was adopted in order to classify injuries (Iona et al., 2021) and is an Italian version of the questionnaire published by Pasulka et al. (2017). The tool includes a preliminary question (“Do you recall any injuries? yes/no”) followed by 12 questions on the history of the injury. The questionnaire defines the following: athletes who have been injured (yes/no), athletes who have had to rest after a sports injury (<1 month of rest from overuse/at least 1 month of rest from serious overuse), and whether athletes had sustained a direct or indirect injury.

#### Physical Well-Being Testing

The Pittsburg Sleep Quality Index (PSQI), 19 items self-reported scale using the “global score” (Buysse et al., 1989) was used to assess the sleep quality of the athletes. The PSQI is the most commonly-used retrospective self-assessment questionnaire, which measures the quality of sleep of an individual during the previous month. The questionnaire assesses seven clinically derived domains of sleep difficulties (sleep quality, sleep latency, sleep duration, habitual sleep efficiency, sleep disturbances, use of sleeping medications, and daytime dysfunction). All of these sleep domains are evaluated as a single factor for overall sleep quality. Usually, an overall score greater than 5 in at least two components indicates a significant sleep disorder, while an overall score of less than 5 in at least three components is an indication of good-quality sleep.

#### Behavioral Testing

The Emotion Awareness Questionnaire (EAQ) (Rieffe et al., 2007) is made up of 30 items and identifies how children or adolescents feel and what they think about their feelings. The current version uses a six-factor structure that describes six aspects of emotional functioning, that is, Differentiating Emotions, Verbal Sharing of Emotions, Bodily Awareness of Emotions, Acting Out Emotions, Analyses of Emotions, Attention to Others’ Emotions. Some items are formulated negatively and therefore have inverse scoring. Participants are asked to rate how true each element is for them on a three point scale (1 = not true, 2 = sometimes true, 3 = often true).

The Multidimensional Assessment of Interoceptive Awareness (MAIA) (Mehling et al., 2012) is composed of 32 items on a 6-point Likert scale, in which the participant has to rate “how often each statement applies to you generally in daily life,” with ordinal responses coded from 0 (“never”) to 5 (“always”). This instrument measures the IAw on the following eight scales: (1) Noticing, which measures the awareness of one’s bodily sensations; (2) Not-distracting, which measures the tendency to avoid ignoring or distracting oneself from sensations of pain or discomfort; (3) Not-worrying, which measures the tendency to avoid experiencing emotional distress or worry with sensations of pain or discomfort; (4) Attention regulation, which measures the ability to sustain and control attention to bodily sensations; (5) Emotional awareness, which measures the awareness of the connection between bodily sensations and emotional states; (6) Self-regulation, which measures the ability to regulate psychological distress by attention to bodily sensations; (7) Body listening, which measures the tendency to actively listen to the body for insight; and lastly (8) Trusting, which measures how much the experiences of one’s body are perceived as being safe and trustworthy.

Awareness Interoceptive Scale (AIS) (Longarzo et al., 2015). To assess IAw and to specifically investigate in what manner and with what frequency subjects feel signals arising from their own body, we used an extended version of the ““How do you feel?” questionnaire” (Camodeca and Rieffe, 2013). The questionnaire included 35 items to be rated on a 5-point Likert scale (0 = never; 1 = sometimes; 2 = often; 3 = very often; 4 = always). The total score ranges from 0–140, with higher scores meaning a higher IAw.

The questionnaires were given on site at each athletic event and took approximately 60 min to complete in a free moment of game performade day.

### Statistical Analysis

The statistical analysis was performed using the software package SPSS 25.0 for Windows.

A descriptive analysis of the sociodemographic characteristics of the study participants was carried out. Descriptive statistics were determined using absolute frequencies and percentages for categorical variables, while for the quantitative variables we used the mean values with standard deviation (SD) and with minimum, medium and maximum values.

The associations among the socio-demographic factors, physical attitudinal characteristics, injuries (recall and description) and psychological-attitudinal aspects were all evaluated. The differences between the results of the various groups—that is, self-referred (yes/no), serious overuse (yes/no) and direct injury (yes/no)—as compared to categorical variables, were analyzed using the Chi-square test or Fisher Exact test. The Odds Ratios (ORs) with corresponding Confidence Intervals at 95% (95% CI) were calculated in order to estimate the risk of injury for dichotomous variables. Three multivariate logistic regression models were established in order to study the outcomes: referred injuries (yes/no), serious overuse (yes/no) and direct injury (yes/no). The inclusion of any covariate in the models was decided on the basis of the univariate analysis (*p* value lower than 0.25). The “stepwise” passage of inferential statistics with backward elimination of non-significant variables (probability to entry p50.05) was subsequently used to generate a minimal model. The suitability of fit for the models was assessed by means of Hosmer and Lemeshow’s test (Lemeshow and Hosmer, 1982). The statistical significance was set at *p* < 0.05.

## Results

### Participants Characteristic

Of the 199 participants enrolled in the study, 169 provided satisfactory data for the analysis and successfully completed the questionnaire. Of these, 38 were female (23%) and 131 male (77%); ages were 11.2 ± 2.7 years, weight was 56.28 ± 15.72 kg, and height was 161.3 ± 15.52cm Table 1 shows the characteristics of the sample.

The athletes engaging in team sports were 139 (82%) and 30 in individual sports; 93% reported training for more than 8 months a year. A high degree of specialization was reported by 49.1% of the athletes (*n* = 169), but only 52% (*n* = 87) of them said they practiced a single sport for fewer than 4 days/week and for more than 3 years (Table 1A).

**TABLE 1A:** Description qualitative variables of the sample.

Qualitative variables		Tot (*N* = 169)	%
Gender	M	131	77
	F	38	22
Educational level	None	15	13
	Elementary school	36	21
	Middle school	69	41
	Secondary school	43	25
Sport	Individual	30	18
	Team	139	82
Years of sport	< 3 years	82	48
	> 3 years	87	52
No. Days of training/week	< 4 days/week	151	89.3
	> 4 days/week	16	9.5
	missing	2	1.2
Months of training	< 8 months	12.0	7.0
	> 8 months	157.0	93.0
Competitions	No	68	40
	Yes	101	60
National competitions	No	71	42
	Yes	98	58
International competition	No	100	59
	Yes	69	41
Specialization	Low	11	6.5
	Medium	75	44.4
	High	83	49.1
Self-referred injury	No	71	42.0
	Yes	98	58.0
Rest from sports for injury	< 1 month of rest from sports (Overuse)	81	82.7
	At least 1 month of rest from sports (serious overuse)	17	17.3
Type of injury	Direct	67	68.4
	Indirect	29	29.6
	Missing	2	2.04
Change in last week	Hours training	49	29.0
	New technique	26	15.4
	New equipment	8	4.7
	Missing	86	50.9

A greater percentage of athletes (58%) reported injuries, with 68.4% (*n* = 67) reporting a direct type of injury and having to stop sports for up to 1 month during the year. Injured athletes had higher sports specialization scores. Self-reported sleep-quality data showed a low value for the PSQI_sum (3.1 ± 1.8) (Table 1B). The emotional aspects of the study showed that all MAIA questions received a high value, particularly in “attention regulation” (16.0 ± 5.8) and “emotional awareness” (12.1 ± 4.7). The total IAw score was average (41.0 ± 19.2), and the eight different evaluation scales can be seen in Table 1B.

**TABLE 1B:** Description quantitative variables of the sample.

Quantitative variables	Tot	Mean	SD	Med	Min	Max
Age (years)	169	11.2	2.7	10.0	7.0	18.0
Weight (Kg)	126	56.3	15.7	56.0	28.0	96.0
Height (cm)	131	161.3	15.5	163.0	127.0	207.0
MAIA _noticing	169	9.3		9.3	0.3	63.0
MAIA_not distracting		3.1	1.3	3.3	0.0	4.7
MAIA _not worrying		3.4	1.7	3.3	0.0	16.0
MAIA _attention regulation		16.0	5.8	15.6	3.3	30.7
MAIA_emotional awareness		12.1	4.7	11.4	1.2	21.0
MAIA_self regulation		8.3	3.3	8.3	0.0	16.3
MAIA_body listening		5.9	2.6	5.7	0.0	11.7
MAIA_trusting		7.3	3.2	7.0	0.0	11.7
PSQI_sleep quality	167	0.6	0.6	0.0	0.0	3.0
PSQI_latency	169	0.6	0.7	0.0	0.0	3.0
PSQI_duration	166	0.3	0.5	0.0	0.0	2.0
PSQI_efficacy	165	0.2	0.5	0.0	0.0	3.0
PSQI_disturbances	169	1.0	0.4	1.0	0.0	2.0
PSQI_hypnotics use	163	0.0	0.3	0.0	0.0	2.0
PSQI_day disturbances	169	0.4	0.6	0.0	0.0	2.0
PSQI_sum	169	3.1	1.8	3.0	0.0	10.0
Awareness Interoceptive Scale (AIS) total score	168	41.0	19.2	38.0	8.0	140.0
Differentiating emotions (EAQ)	143	2.0	0.4	2.0	1.0	3.0
Verbal sharing of emotions (EAQ)		2.1	0.5	2.0	1.0	3.0
Not hiding emotions (EAQ)		2.0	0.5	2.0	1.0	3.0
Bodily awareness of emotions (EAQ)		2.0	0.4	2.0	1.0	3.0
Attending to others’ emotions (EAQ)		2.1	0.4	2.0	1.0	3.0
Analyses of (own) emotions (EAQ)		2.0	0.4	2.0	1.0	2.8

### Correlation Analyses Data

The univariate analysis, adjusted for gender, type of sport, national and international competitions, specialization and years of attendance in sports, showed that high specialization for “reported injuries” is not a risk factor, but the difference was not statistically significant (82%, *p* = 0.214). The anthropometric variables such as age (*p* = 0.007), weight (*p* = 0.007) and height (*p* = 0.007) were significant in the cases of self-referred injuries (self-referred traumas, not those derived from the patient’s records) while these athletes had significant mean PSQI scores of 3.1 ± 1.8; *p* = 0.007 (Table 1B). Three variables of Emotional awareness were also noteworthy, namely “body awareness of emotion” (EAQ) (*p* = 0.007), “attending to others” emotions’ (EAQ) (*p* = 0.012) and “analyses of emotion” (EAQ) (*p* = 0.016). Only the variable “verbal sharing emotion” (EAQ) (*p* = 0.002) was significant in the cases of rest from sport because of injury. Regarding the type of injury, the MAIA variables “noticing (*p* = 0.061) and not distracting” (*p* = 0.016) are as significant as those of “sleep quality” (PSQI) (*p* = 0.050).

The athletes who had been training for more than 3 years (70%, OR = 2.5; *p* = 0.003) or that participate in national competitions (70%, OR = 2.5; *p* = 0.005) turned out to be more exposed to risk. In team sports (100%, OR = 0.75; *p* = 0.022) a high risk of having to remain at rest for up to 1 month because of overuse was observed. Males who suffered direct trauma (70%; OR = 1.03; *p* = 0.006) in the team group (95%, OR = 0.09; *p* = 0.008) were more exposed to that type of injury, and also the type of specialization figured significantly (*p* = 0.047) (Table 2).

**TABLE 2:** Univariate analysis in “reffered injury,” “rest from sport for injury” and “type of injury”.

Variables		Referred injures				Rest from sports for injury				Type of injures			
		No	Yes	P*	OR	< 1 month of rest from sports (Overuse)	> 1 month of rest from sports (serious overuse)^	p*	OR	Indirect	Direct	*p* *	OR
		*N* (%)	*N* (%)			*N* (%)	*N* (%)			*N* (%)	*N* (%)		
Gender	*F^*	11 (29)	27 (71)	**0.064**	0.48	21 (78)	6 (22)	0.432	0.64	19 (70)	8 (30)	**0.006**	1.03
	*M*	60 (46)	71 (54)			60 (84)	11 (16)			48 (70)	21 (30)		
Sport	Individual	61 (44)	78 (56)	0.288	1.56	61 (78)	17 (22)	**0.022**	0.75	49 (64)	28 (36)	**0.008**	0.09
	Team^	10 (33)	20 (67)			20 (100)	0 (0)			18 (95)	1 (5)		
national competition	No^	39 (55)	32 (45)	**0.005°**	2.51	28 (87)	4 (12)	0.378	1.71	24 (75)	8 (25)	0.432	1.46
	Yes	32 (33)	66 (67)			53 (80)	13 (20)			43 (67)	21 (33)		
international competition	No^	45 (45)	26 (38)	0.428	1.35	45 (82)	10 (18)	0.805	0.87	37 (67)	18 (33)	0.534	0.75
	Yes	55 (55)	43 (62)			36 (84)	7 (16)			30 (73)	11 (27)		
Specialization	Low	2 (18)	9 (82)	0.214	-	8 (89)	1 (11)	0.271	-	8 (89)	1 (11)	**0.047**	-
	Medium	32 (43)	43 (57)			38 (88)	5 (12)			24 (57)	18 (43)		
	High	37 (45)	46 (55)			35 (76)	11 (24)			35 (78)	10 (22)		
> 3 training years	No^	44 (54)	38 (46)	**0.003**	2.57	3 (84)	6 (16)	0.746	1.20	30 (81)	7 (19)	0.056	2.54
	Yes	27 (31)	60 (69)			49 (82)	11 (18)			37 (63)	22 (37.3)		

*P-value of Chi-square test.

*°p-value Fisher’s Exact test.*

*Bold p < 0.05.*

*^reference group.*

### Multivariate Logistic Regression Models

In the logistic model the athletes who had had more than 3 years of training had a 19.79 times greater risk (95% CI: 3.38–116.1) as a “self-referred injury.” An higher risk of self-referred injury was also shown in those who have belonged to the “medium -specialized “category (OR = 7.61; 95% CI: 1.4–41.4), who was more sensitive to the emotions of others” (attending to other’s emotions (EAQ)]” (OR = 42.21; 95% CI: 3.6-487.9), who haven’t a high “bodily awareness of emotions” score (Table 3A).

**TABLE 3A:** Regression model of “Reffered injures”.

Covariates	Reffered injures		
	OR	CI 95%	
		**Inf**	**sup**
Gender (M/F^)	0.14	0.01	1.56
Age (years)	1.14	0.71	1.84
Weight (Kg)	0.98	0.88	1.09
Height (cm)	0.97	0.91	1.05
National competition (Y/N^)	1.37	0.76	2.4
> 3 training years (Y/N^)	**19.79**	**3.38**	**116.1**
High specialization (Y/N^)	1.28	0.03	48.08
Middle specialization (Y/N^)	**7.61**	**1.4**	**41.45**
MAIA not worrying	0.95	0.68	1.33
PSQI	0.82	0.46	1.47
Verbal sharing of emotions (EAQ)	0.27	0.04	1.71
Not hiding emotions (EAQ)	1.05	0.091	12.1
Bodily (abod) awareness of emotions (EAQ)	**0.02**	**0.001**	**0.34**
Attending to others’ emotions (EAQ)	**42.21**	**3.65**	**487.95**
Analyses of (own) emotions (EAQ)	1.37	0.16	11.54
Awareness Interoceptive Scale (AIS) total score	1.01	0.97	1.05
Hosmer and Lemeshow test *p*-value	0.456		

*Bold: p < 0.05.*

*^reference group.*

Concerning the regression model of risk of “rest from sports for injury,” there aren’t significant association (Table 3B).

**TABLE 3B:** Regression model of “Rest from sports for injury”.

Covariates	Rest from sports for injury		
	OR	CI 95%	
		inf	sup
*MAIA not distracting*	0.64	0.41	1.00
*Verbal sharing of emotions (EAQ)*	0.36	0.05	2.36
*Bodily awareness of emotions (EAQ)*	0.20	0.04	1.11
*weight (Kg)*	0.95	0.91	1.00
*PSQI*	1.36	0.90	2.07
Hosmer and Lemeshow test *p*-value	0.300		

*Bold: P < 0.05.*

The third model of regression analysis (Table 3C) showed that the athletes who had more risk of sustaining a direct trauma was those had than 3 years of training (OR = 4.60; 95% CI: 1.06–19.9) and athletes having a high level of “analyses of emotion”: OR = 5.38 95%CI: 1.18–24.57. On the contrary A protective effect was shown in who was high score of MAIA distracting (OR = 0.59; 95%CI:0.38–0.91).

**TABLE 3C:** Regression model of “Type of injury, direct”.

Covariates	Type of injury direct		
	OR	CI 95%	
		inf	sup
Gender (M/F^)	1.50	0.26	8.57
Weight (Kg)	0.98	0.93	1.03
>3 training years (Y/N^)	**4.60**	**1.06**	**19.94**
High specialization (Y/N^)	0.42	0.12	1.46
Middle specialization (Y/N^)	1.12	0.05	24.13
MAIA trusting	1.13	0.89	1.44
MAIA distracting	**0.59**	**0.38**	**0.91**
PSQI	1.45	0.99	2.12
MAIA noticing	0.96	0.89	1.04
Not hiding emotions (EAQ)	2.20	0.41	11.80
Bodily awareness of emotions (EAQ)	0.25	0.06	1.01
Analyses of (own) emotions (EAQ)	**5.38**	**1.18**	**24.57**
Hosmer and Lemeshow test *p*-value	0.476		

*Bold: P < 0.05.*

*^reference group.*

## Discussion

Although some studies have suggested that regardless of the age and amount of training of young athletes, sports specialization alone can increase the overall risk of serious injury from overuse, our data suggests that specialization in a single sport or early specialization in the sport cannot be considered factors of risk for young athletes. Moreover, defining sports specialization along a continuum (low, moderate, high) may be useful for studying and quantifying the risk of injury.

*Independent risk of injury in young athletes specializing:* The first descriptive analysis that we carried out showed that 93% of team athletes have been training for more than 3 years and that 49% are highly specialized because they have been training for more than 3 years, more than 4 days a week. Non-parametric univariate analysis showed that high specialization is not a risk factor for reported injuries. In the present study, mid-level athletes showed a greater risk of injuries, even serious ones due to overuse, equal to those who have been training for more than 3 years, while the risk is lower for athletes practicing individual sports. Several authors have argued that there is an independent risk of injury in young athletes specializing in a single sport (Jayanthi et al., 2013; Pasulka et al., 2017) as well as in elite young athletes (Moseid et al., 2017). Since the self-reported age of specialization is similar for all involved subjects, it is not possible to determine whether the risk of injury is different for those who specialized at a younger age than for those at an older age. Long-term longitudinal studies are needed in which a younger population of specialized athletes is compared to diversified athletes in order to determine whether there is specific starting age that could reduce the risk of injury (Jayanthi et al., 2013).

*Physical well-being is related to referred injury and direct sport type:* Based on the data, sleep (Fullagar et al., 2015; Swinbourne et al., 2016) was a biological variables to be linked to the emotional and psychological status of the athletes. Experiencing poor sleep quality has important implications including potentially impaired physiological and cognitive recovery, increased physical and mental health consequences, an increased risk of injury, and compromised performance outcomes (Juliff et al., 2015). The sleep health of individual- and team-sports athletes is generally poorly understood, including sleep quality and sleep duration, and how their functioning is affected outside the laboratory in the context of psychological, emotional, and physical demands that are present during a competitive season. Current data show that athletes generally sleep well, but the PSQI_sum is related to referred injury and direct sport type. Regularly obtaining PSQI_sum results scores of less than 5 means a 1.3 times greater risk for direct injury than athletes who have reported “referred injuries” or by injuries resulting from overuse. These findings suggest that the additional factors associated with sports specialization increase the risk of injury.

*IAw has been shown to protect against overuse and severe overuse injuries*: It has been reported in literature that significant psychological factors capable of predicting the occurrence of sports injuries include the following: somatic trait anxiety (Li et al., 2017), mistrust (Johnson and Ivarsson, 2011), negative life-event stress and negative coping abilities (Lathlean et al., 2019). However, it must be noted that the investigation of an athlete’s psychological-attitudinal-emotional status is a new field of research (Erden and Emirzeoğlu, 2018). Interoception research typically distinguishes between interoceptive sensitivity (IS) and interoceptive awareness (IAw). Interoceptive sensitivity (IS) describes the objective detection of internal bodily sensations through behavioral measures, such as heartbeat perception tasks, while interoceptive awareness (IAw), refers to self-reported detection of internal bodily sensations using questionnaire-based measures (Garfinkel et al., 2015). Our descriptive analyses revealed significant scores on two of the eight MAIA scales, namely Emotional awareness and Attention regulation, which were high. Linear regression analyses further clarified the specific contribution of each MAIA scale by explaining the variability between participants. High scores on the “Not worrying” (tendency not to worry or experience emotional pain in the presence of physical pain) or “feeling of discomfort” sections on the MAIA scale was a protective factor toward the self-referred injury and was linked to “Attending to others’ emotions” (EAQ). Thus, participants who reported being more prone to feeling emotional distress or worry in response to negative body sensations (i.e., with a low score in the “Not worrying” scale) also reported being more likely to pay attention to others’ emotions (“Attending to others’ emotions**”**—EAQ), as the highest scores show. This result suggests there is a close connection between these two constructs, which makes sense, because both are related to measures of emotion. A high score on the MAIA variable “not distracting” (Tendency to avoid ignoring or distracting oneself from feelings of pain or discomfort) reduced by half the probability of having to stop engagement in sport for more than 1 month due to injury.

The third model of regression analysis showed that the athletes who had more risk of sustaining a direct trauma was those had than 3 years of training and athletes having a high level of “analyses of emotion risk” EAQ. The “Analyses of (one’s own) emotions” variable had a higher score for the type of injury, therefore those who knew their emotions well were more at risk of direct injury. Research on health, social, and sport-injury suggests that emotional awareness support has the potential to moderate those psychological responses to injury that are detrimental to athletes’ health and well-being. Despite growing empirical research on the role of stress and psychological distress in sports injuries, there is a lack of study on emotional awareness and interoceptive functions in relation to responses to injury. Also in this case MAIA not distracting showed a protective effect in who was high score of direct trauma. The tendency to avoid ignoring or distracting oneself from feelings of pain or discomfort confirm a positive influence on the type of injury. Highlighted aims discussed in the latest and most recent research (Jayanthi et al., 2022), the present work provides support for the functional aspects of the prevention of injury-related stress factors and is important for identifying possible interventions that might be useful for accelerating a positive return to sport in injured young athletes. It also has important implications for interventions that could aim at expediting an injured young athlete’s successful return to sport. Currently, there are no guidelines or recommendations for athletic trainers/therapists to address psychological and emotional factors with the purpose of injury prevention. All these factors can and should be discussed with one’s local sport technical and medical staff.

### Limitations

Our study has several limitations. It is not population-based and therefore cannot provide real incidence rates for injuries in highly-skilled versus moderately-skilled versus low-skilled athletes, particularly in non-Italian populations. When defining sports specialization using the three-point scale, it is important to take into account those athletes who have only participated in one sport. The wide variety of sports reported by participants in the present study does permit the inclusion of sport type in regression models. Since only self- referred injuries have been included, the number of overuse injuries could be an underestimation. The association between overuse injury and specialization and training in specific sports needs to be further evaluated in order to provide sport-specific recommendations for injury prevention. The specific psychological factors of burnout, anxiety and pressure were not evaluated in our study but the fact that they do interact with the incidence of injury in specialized young athletes is suggestive.

## Conclusion

This study shows that, even though young athletes seem to be at a high risk of becoming injured, early team sport specialization and a high performance level cannot be considered to be the only risk factors. It is recommended that medicine teams and coaches be observant the athletes’ behavior in terms of emotional, awareness interoception, elevated anxiety levels and sleep quality and duration. This is especially important for clinicians, coaches, and parents when counseling young athletes on the risks of overuse injuries related to sports participation. Longitudinal studies that follow large cohorts of athletes in each type of sport are necessary in order to better understand the relationships between sports specialization, sports type, injury risk and emotional and interoceptive status.

## Data Availability Statement

The raw data supporting the conclusions of this article will be made available by the authors, without undue reservation.

## Ethics Statement

The studies involving human participants were reviewed and approved by Local Ethics Committee Azienda Ospedaliera Universitaria Mater Domini. Written informed consent to participate in this study was provided by the participants’ legal guardian/next of kin.

## Author Contributions

GL, AM, DM, and TI conceived and designed the experiment, selected the participants, and collected data. PT, DC, AA, SR, and TI analyzed data. AM, GL, DM, DC, SR, and TI wrote the original draft. All authors have read and agreed to the published version of the manuscript.

## Conflict of Interest

The authors declare that the research was conducted in the absence of any commercial or financial relationships that could be construed as a potential conflict of interest.

## Publisher’s Note

All claims expressed in this article are solely those of the authors and do not necessarily represent those of their affiliated organizations, or those of the publisher, the editors and the reviewers. Any product that may be evaluated in this article, or claim that may be made by its manufacturer, is not guaranteed or endorsed by the publisher.
